# The Role of NK Cells and Innate Lymphoid Cells in Brain Cancer

**DOI:** 10.3389/fimmu.2020.01549

**Published:** 2020-07-31

**Authors:** Alexander James Sedgwick, Nazanin Ghazanfari, Patrick Constantinescu, Theo Mantamadiotis, Alexander David Barrow

**Affiliations:** ^1^Department of Microbiology and Immunology, The University of Melbourne and The Peter Doherty Institute for Infection and Immunity, Melbourne, VIC, Australia; ^2^Department of Surgery, The Royal Melbourne Hospital, The University of Melbourne, Parkville, VIC, Australia

**Keywords:** clinical trial, immunotherapy, brain cancer, innate lymphoid cell, NK cell

## Abstract

The brain is considered an immune privileged site due to the high selectivity of the blood-brain barrier which restricts the passage of molecules and cells into the brain parenchyma. Recent studies have highlighted active immunosurveillance mechanisms in the brain. Here we review emerging evidence for the contribution of innate lymphoid cells (ILCs) including natural killer (NK) cells to the immunosurveillance of brain cancers focusing on glioblastoma, one of the most aggressive and most common malignant primary brain tumors diagnosed in adults. Moreover, we discuss how the local tissue microenvironment and unique cellular interactions influence ILC functions in the brain and how these interactions might be successfully harnessed for cancer immunotherapy using insights gained from the studies of autoimmunity, aging, and CNS injury.

## Introduction

The global age-adjusted incidence of brain tumors is 5.57 per 100,000 people with over half being astrocytic tumors (1). Of astrocytomas, the most malignant form, glioblastoma (GBM), is diagnosed at a much higher frequency than lower grade astrocytomas. Even with an aggressive treatment regime, comprised of maximal safe resection, radiotherapy, and administration of the DNA alkylating agent, temozolomide (TMZ), the mean GBM survival time ranges between 12 and 15 months. Whilst recent advances in cancer immunotherapy have enhanced expectations for improved patient outcomes, current GBM treatment options remain limited and the mean overall survival of GBM patients has failed to improve over the last decade. The unique anatomy of the brain, the exclusive nature of the blood-brain barrier (BBB), and a poorly immunogenic, complex, and immunosuppressive tumor microenvironment (TME) represent major challenges in treating malignant brain cancers. Here, we review emerging evidence for brain tumor immunosurveillance by NK cells and ILC subsets.

### Natural Killer Cells

**Figure 1:**
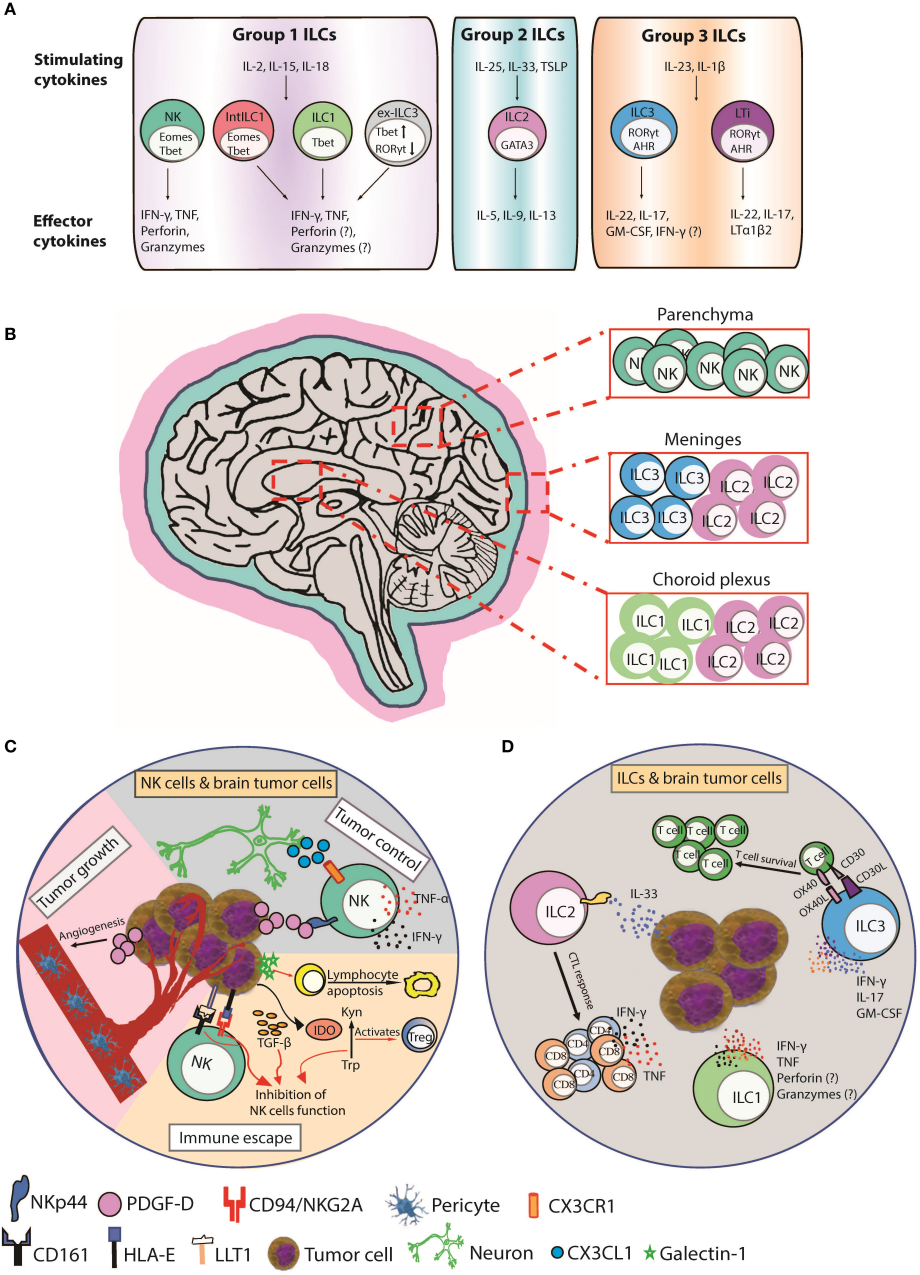
Function, distribution, and anti-tumor responses of CNS ILC subsets. **(A)** Group 1 CNS ILCs have thus far been shown to include NK cells, intermediate ILC1s (intILC1s), ILC1s, and “ex-ILC3s.” NK cells express T-bet and Eomes and secrete IFN-γ and TNF in response to IL-2, IL-15, and IL-18, and lyse malignant cells via perforin, and granzymes. ILC1s express T-bet and produce IFN-γ and TNF in response to IL-2, IL-15, and IL-18 to promote type I immunity. Intermediate ILC1 (IntILC1) represent an intermediate phenotype between NK and ILC1 and express T-bet and Eomes. Ex-ILC3s are former ILC3 that have upregulated T-bet and downregulated RORγt to differentiate into ILC1-like cells. ILC2s express GATA3 and secrete IL-5, IL-9, and IL-13 in response to IL-25, IL-33, and TSLP to promote type II immunity. Group 3 ILCs include ILC3s and LTi cells. ILC3s express RORγt and AHR and produce IL-17, IL-22, and GM-CSF in respond to IL-23 and IL-1β stimulation to counteract extracellular bacterial and fungal infections. LTi cells also express RORγt and AHR and produce IL-17, IL-22, and lymphotoxin (LTα1β2). LTi cells trigger lymphoid tissue organogenesis during development. **(B)** NK cells are mainly present in the brain parenchyma. ILC1s are enriched in choroid plexus, whereas ILC2s accumulate in the choroid plexus and meninges and ILC3s accumulate in the meninges. **(C)** CX3CR1^+^ NK cells can infiltrate the brain in response to CX3CL1 chemokine produced by neurons. (Tumor control) PDGF-D expressed by tumor cells binds to the activating NKp44 receptor expressed on activated NK and induces the secretion of IFN-γ and TNF that inhibits tumor cell proliferation. (Tumor growth) PDGF-D enhances tumor growth by promoting pericyte recruitment and tumor angiogenesis. (Immune escape) Tumor cells upregulate IDO, which inactivates NK cells and activates immunosuppressive regulatory T cells (Tregs) by depletion of Trp and accumulation of Kyn. Tumor cells also secrete galectin-1 that induces lymphocyte apoptosis. Tumor cells supress NK cells function by inducing HLA-E and LLT1 ligands, which are ligands for NK cell inhibitory receptors CD94/NKG2A and CD161, respectively. TGF-β also inhibits NK cells function. **(D)** A hypothetical scheme showing the possible role of ILCs in brain cancer. Human ILC1s and ILC3s can express NKp44 and secrete IFN-γ and/or TNF in response to PDGF-DD to promote anti-tumor immunity (2). ILC2s enhance CTL responses to control the spread of tumors in response to IL-33 produced by tumor cells. ILC3s can also produce proinflammatory cytokines IFN-γ, IL-17, and GM-CSF and express the costimulatory molecules CD30L and OX40L that can promote T cell survival and function. AHR, aryl hydrocarbon receptor; CTL, cytotoxic T lymphocyte; EOMES, eomesodermin; GATA3, GATA-binding protein 3; GM-CSF, granulocyte-macrophage colony-stimulating factor; IDO, indoleamine 2,3-dioxygenase; Kyn, kynurenine; LLT1, Lectin-like transcript-1; LTi, Lymphoid tissue-inducer; PDGF-D, Platelet Derived Growth Factor D; RORγt, retinoic acid-related orphan receptor gamma t; T-bet, T-box expressed in T cells; Trp, tryptophan; TSLP, thymic stromal lymphopoietin.

NK cells are large granular lymphocytes considered the innate counterparts of cytotoxic T lymphocytes (CTL) due to their spontaneous ability to lyse malignant and virus-infected cells, whereas ILC1, ILC2, and ILC3 mirror adaptive T helper subsets (Figure 1A). NK cells respond to “stressed” cells that downregulate MHC class I (MHCI) to evade CTL recognition and are therefore critical for anti-tumor immunity whenever CTL are compromised (3). NK cells are present at lower frequencies in the brains of naïve mice (4) (Figure 1B), but during neuropathological conditions, such as virus infection or autoimmunity, the BBB can become permeable, allowing NK cell migration into the CNS (5–9).

### NK Cells in Brain Cancer

The CX3CL1 chemokine (also known as fractalkine) produced by neurons mediates CX3CR1^+^ NK cell recruitment to the brain and is associated with a favorable glioma prognosis (10) (Figure 1C). In one study, NK cells constituted the highest percentage of lymphocytes infiltrating GBM compared to breast cancers or melanomas suggesting a prominent role for NK cells in brain cancer surveillance (11). NK cells infiltrate meningiomas and metastatic brain neoplasms (12–14) and lyse GBM and medulloblastoma tumor cell-lines *in vitro* (15–22). NK cell receptors are linked with brain tumor surveillance and an allele of the activating KIR2DS4 receptor is associated with control of cytomegalovirus (CMV)-positive GBMs (23). CMV-induced expression of platelet-derived growth factor D (PDGF-D) enhanced GBM growth by promoting pericyte recruitment and tumor angiogenesis (24) (Figure 1C). PDGF-D is expressed by most GBMs and binds to the activating NKp44 receptor to stimulate cytokine secretion from NK cells and ILCs to control tumor growth, which was associated with improved survival of GBM patients (2) (Figure 1C). These studies implicate NK cells engage in brain tumor surveillance that impacts prognosis (14).

Several computational-based studies show that glioma patients expressing activated NK cell transcriptional signatures (TS) have improved prognosis (2, 25–29). Studies in patients and mouse models support these findings (10, 30, 31), with one human study showing a remarkable relationship between the presence of activated NK cells and improved survival in GBM (32). Another study showed that activated NK cells were higher in low grade compared to high grade gliomas suggesting reduction in activated NK cells is associated with transition from low to high grade brain cancers (27). NK cells may therefore play a detrimental role in brain tumor progression and heterogeneity. Expression of B7-H6, a ligand for the activating NKp30 receptor, is elevated in human glioma and associated with tumor progression (33). Whilst NK cells efficiently lyse undifferentiated GBM cancer stem cells (CSC), NK cell-derived IFN-γ promotes GBM CSC differentiation and decreased susceptibility to NK cell cytotoxicity (34, 35). In GBM, CSCs that survive therapy are a source of tumor recurrence/relapse. Influencing the balance of NK cell-mediated lysis of CSCs or opposing the pro-tumorigenic effect of NK cell-IFN-γ-induced CSC differentiation will be an important mechanism to decipher and target. Interestingly, IFN-γ-induced CSC differentiation concomitantly enhances tumor susceptibility to chemotherapy, suggesting NK cell-based therapies can be combined with other therapeutic strategies for more effective clinical outcomes (36, 37).

### NK Cell-Based Immunotherapies for Brain Cancer

CNS tumors are often poorly immunogenic and highly immunosuppressive which imposes barriers to successful immunotherapy (38). A summary of current research and clinical trials into NK cell immunotherapies for malignant CNS tumors is provided (Table 1). Whilst NK cell cytotoxicity is facilitated by an array of activating receptors (62, 63) the chief inhibitory signal for NK cells, MHC class I (MHC-I), can be overexpressed in CNS malignancies and suppresses NK cell activity (64, 65). Strategies to enhance NK cell anti-tumor function include activating the DNA damage response (DDR) to induce ligands for activating NK cell receptors (66–68). The proteosomal inhibitor bortezomib (BTZ) activates the DDR and sensitizes GBM cells to NK cell killing by inducing ligands for the activating receptors, NKG2D (39, 59, 69, 70) and DNAM-1 (40). BTZ treatment with autologous NK cells suppressed tumor growth and prolonged survival in 25% of test animals (70). However, appropriate BTZ scheduling with NK cell transfer remains to be optimized to prevent sensitization of NK cells themselves (70). GBM patients have increased expression of NKG2D ligands (NKG2DL) following TMZ therapy and TMZ-induced activation of the DDR improved survival in a mouse model of GBM that was NKG2D-dependent (41).

**Table 1:** Summary of current academic investigations and clinical trials into NK cell immunotherapy directed against malignant tumors of the CNS.

**Therapeutic Strategy**	**Treatment**	**Results summary**	**Model (tumor type)**	**Reference/Trial ID**
Combinational therapy NK cells and chemotherapy	NK cells infusion with: Sulindac; MAPK and cyclin-dependent kinase 4/6 inhibitors (38)	Reduces VEGF secretion and increases NK cell cytotoxicity; Suppresses tumor proliferation and increases NK cell cytotoxicity	Human (GBM, Lung cancer)	(37, 38)
Sensitization of tumors to NK cell cytotoxicity	NK cell infusion with BTZ	Predisposes tumor to NK natural cytotoxicity and TRAIL/DR5; BTZ and NK cell infusion increased tumor elimination	Mouse (BG7); mouse (U87)	(39, 40)
Virotherapy	Triple therapy (NK cell infusion, BTZ, oHSV)	Tumor clearance tumor bearing mice; combinational therapy with BTZ and oHSV enhances tumor death and NK cell activation	Mouse (GBM30)	(41, 42)
	TGF-β and oHSV infection	Modulated NK cell immune response to oHSV infected cells and improved anti-GBM effect of the oHSV treatment	Mouse (GB30)	(43)
Overcoming immunosuppressive TME	siRNA interference of TGF-β signaling; infusion of TGF-β receptor negative NK cells	Reduction of tumorigenic profile of glioma; NK cells were resistant to TGF-β inhibition	Mouse (LNT229); *in vitro*; *in vitro*	(44–46)
Toll-like receptor agonism	CpG-ODN DNA TLR-9 agonism	Clinical trials failed to recapitulate results of encouraging *in vitro* TLR-9 agonism	Clinical trial (GBM); mouse (GL621)	(47–50)
	Poly-ICLC TLR-3 agonism with bevacizumab (a-VEGF)	Poly-ICLC adjuvant to tumor associated antigens mixed with Bevacizumab—study unfinished, promising *in vitro* results	Human trial (GBM)	NCT02754362 (withdrawn—personnel changes)
Directing NK cell immunity toward brain tumor antigens	Infusion of monalizumab (a-NKG2A)/cetuximab (a-EGFR)	NKG2A blockage potential to boost ADCC against GBM. Cetuximab treatment increased ADCC mediated by CD16+ NK cells (IL-2 or lectin-activated)	Human GBM stem cells *in vitro*	(51)
	Infusion of CAR NKs engineered to be specific for EGFR, EGFRVIII, Erbb2	EGFR, EGFRvIII CAR NK cells suppressed tumor growth and significantly prolonged survival; CXCR4 transduction enhanced antitumor responses of EGFR CAR NK cells; Erbb CAR NK cells	Mouse (GB19 xg); mouse (U87 xg); mouse (GL621 xg)	(52–54)
	Infusion of a-NG2/CSPG4 Ab	NK cell directed ADCC and proinflammatory tumor environment enhancing survival	Rat (U87); mouse (GBM)	(55, 56)
Immune checkpoint blockade	Infusion of a-PD-1 and a-CTLA-4 Ab	Interference with peripheral immune cell inhibition potentiates intracranial immune response; immune checkpoint blockade antibodies improved survival in models	Mouse(GL621); mouse (B16)	(57, 58)
	Blockade of interactions of HLA-E:CD94/NKG2A or LLT1/CD161 with siRNA or blocking Ab	Blockade promoted NK cell lysis	Human (GBM) *in vitro*	(59, 60)
Circumventing the BBB	Infusion of a-CTLA-4/a-PD-1 Ab conjugated to biopolymer scaffold	Treatment able to cross BBB. Prolonged survival of mice compared to free a-CTLA-4 and a-PD-1 Ab	Mouse (GL261)	(61)
Autologous NK cell infusion expanded *ex vivo*	Artificial antigen-presenting cells	Promising *in vitro*, results forthcoming	Human trial (Recurrent MBM)	NCT02271711 (active)
	Cytokines/Feeder cells + infusion recombinant human interleukin-15	Promising *in vitro*, results forthcoming	Human (Solid brain tumors, SCM, NBM)	NCT01875601 (complete)
	Cancer/testis antigens presented by DNA-demethylated T_H_ cells	Labeled cells infiltrated tumor site (SPECT imaging). Reduced disease burden 5 out of 10 patients. Long term survival in 3 of 10. However, slow kinetics of induced antitumor response	Human trial (GBM)	NCT01588769 (complete)
	Genetically modified feeder-cells	Promising *in vitro*, results forthcoming	Human trial (GBM)	NCT04254419 (not yet recruiting)
Allogeneic (HLA)-haploidentical hematopoietic cell transplantation	Allo HTC and infusion with donor NK cells	Promising *in vitro*, results forthcoming	Human trial (eSCM, rSCM, oSCM, NBM)	NCT02100891 (recruiting)

*Abbreviated cancers: GBM, glioblastoma; MBM, medulloblastoma; NBM, neuroblastoma; e, Ewing; SCM, sarcoma; r, Rhabdomyosarcoma; o, Osteosarcoma. Other abbreviations: xg, Xenograft; VEGF, vascular endothelial growth factor; BTZ, bortezomib; oHSV, oncolytic herpes simplex virus; TGF-β, transforming growth factor-beta; EGFR, epidermal growth factor receptor; Ab, antibody*.

Anti-cancer treatments, such as chemotherapy, selective inhibitors of oncogenic signaling pathways, or oncolytic virotherapy can promote tumor cell death and enhance immunogenicity, which holds great potential when combined with immunotherapy (42, 71). Using a triple therapy approach, BTZ treatment combined with an oncolytic Herpes Simplex Virus (oHSV) strain sensitized GBM to adoptive NK cell therapy (40). BTZ enhanced expression of ligands for activating NK cell receptors, such as DNAM-1, whilst oHSV infection induced NK cell secretion of IFN-γ and TNF, which enhanced tumor cell death and improved survival of athymic nude mice transplanted with GBM tumors (40). Conversely, other studies claim NK cells limit oncolytic virotherapy by curbing virus infection of tumor cells. Transient TGF-β delivery or NK cell depletion increased oHSV titers, suppressed tumor growth, and prolonged survival in mouse GBM models (43, 72). TGF-β is considered a pro-tumor cytokine (73, 74) that suppresses NK cell function by downregulating activating NK cell receptors (75–77) or their ligands on brain tumor cells (78) (Figure 1C), reducing NK cell proliferation and converting NK cells into pro-tumor ILC1-like cells (79), or upregulating immunosuppressive extracellular matrix TME components, such as the galectins (44). Neutralizing TGF-β in the brain TME rescued NK cell anti-tumor function in glioma or medulloblastoma patients (45, 46) and expression of a dominant negative TGF-β receptor lacking the kinase domain (80) restored NK cell cytotoxicity against GBM and medulloblastoma cells in the presence of TGF-β *in vitro* (46, 81).

Brain tumors also secrete other soluble immunosuppressive factors, such as the carbohydrate-binding protein, galectin-1, that reduced lymphocyte viability (82) (Figure 1C). Galectin-1-deficient gliomas are more susceptible to NK cell lysis and were eradicated by NK cells before adaptive anti-tumor immune responses (17). Alternatively, glioma cells upregulate indoleamine 2,3-dioxygenase (IDO), a key rate-limiting enzyme of tryptophan (Trp) metabolism. IDO is involved in tumor-derived immunosuppression through Trp depletion and accumulation of the metabolite kynurenine that inactivated NK cells and promoted immunosuppressive regulatory T cells (Tregs) (83) (Figure 1C).

Brain tumor cells also modulate their cell-surface to suppress NK cell function. Gain-of-function mutations in isocitrate dehydrogenases (IDH1 and IDH2) in diffuse gliomas promotes epigenetic reprogramming of a number of immune genes including NKG2DL downregulation and resistance to NK cell-mediated lysis (60). Decitabine (a hypomethylating compound) increased NKG2DL expression and restored NK cell-mediated lysis of IDH mutant cells in an NKG2D-dependent manner. In addition to downregulating activating NK cell surface interactions, brain tumors also promote inhibitory NK cell surface interactions. Malignant gliomas induce HLA-E or Lectin-like transcript-1 (LLT1), which can induce NK cell inhibition by binding CD94/NKG2A and CD161, respectively (51, 84–86) (Figure 1C). Blockade of HLA-E:CD94/NKG2A or LLT1/CD161 inhibitory interactions using small interfering RNA or blocking antibodies promoted NK cell lysis of glioma cells (65, 87). Interestingly, a humanized anti-NKG2A antibody, “monalizumab,” in combination with the anti-epidermal growth factor receptor (EGFR) “cetuximab,” is effective in promoting antibody-dependent cellular cytotoxicity (ADCC) against cetuximab-coated head and neck squamous carcinoma (88). EGFR is a prime target for therapy across a broad variety of tumor types including gliomas, suggesting NKG2A blockade with monalizumab has potential to boost NK cell-mediated ADCC against gliomas, particularly those resistant to TMZ (89).

Cancer adjuvants provide other means of reinvigorating anti-tumor immune responses. Oligodeoxynucleotides containing unmethylated cytosine-guanosine motifs (CpG-ODNs) mimic pathogen-associated molecular patterns (PAMPs) that bind to Toll-like Receptor 9 to induce type-I interferon (IFN-I) production from plasmacytoid dendritic cells (pDCs) (90). IFN-I enhances NK cell anti-tumor functions (47, 48) and CpG-ODN stimulation and Treg ablation unleashed NK cell cytotoxicity toward intracranial tumors (49). Despite encouraging results *in vitro*, human trials of CpG-ODN treatment in patients with primary (50) and recurrent GBM (52, 91) reported no benefit in CpG-ODN therapy, suggesting combination treatments are necessary, e.g., Treg depletion that may improve clinical responses. Intriguingly, depletion studies have implicated NK cells as the predominant anti-tumor effector cell in murine models of glioma following repeated low dose administration of CpG-ODN (53). However, tumor-infiltrating NK cells remained susceptible to suppression both locally and systemically, reinforcing the need for more effective methods of augmenting NK cell function in the brain TME.

Chimeric Antigen Receptors (CARs) are tumor-specific antibody single-chain variable fragments (scFvs) fused by a transmembrane linker domain to the CD3ζ signaling chain of the T cell receptor that can be transduced into autologous cytotoxic T lymphocytes (CAR T) or NK cells (CAR NK), respectively (54). CAR NK cells are attractive because they can be engineered to respond to a tumor antigen whilst retaining capacity for natural cytotoxicity. CAR NK cells directed toward EGFR or the constitutively activated mutant EGRFvIII GBM tumor antigen (55, 56) and ErbB2 (92) have shown potent cytotoxicity toward primary GBM tumor cells and cell lines *in vitro*. Some studies targeting GBM antigens show NK cells are important regulators of proinflammatory environments through IFN-γ secretion rather than cytotoxicity (57, 58). Transfer of NKG2D-CAR T cells combined with radiotherapy exhibited therapeutic synergy in mice bearing orthotopic tumors of the murine glioma cell line, GL261, although mice receiving intratumoral vs. intravenous CAR T cells were more likely to survive, reiterating the poor infiltration of intravenously administered CAR T cells into the brain parenchyma (61). Such studies highlight the need to develop immunological and mechanical adjuvants in concert with NK cell therapy to improve delivery to the brain parenchyma.

The BBB is a selectively permeable membrane that excludes harmful material from the parenchyma but impedes delivery of immunotherapeutic agents and cells to brain tumors. Anti-PD-1 and anti-CTLA-4 checkpoint blockade therapy increased NK cell and CD8^+^ T cell infiltration to the CNS and improved survival in models of GBM and melanoma brain metastases (93, 94). The conjugation of poly(β-L-malic) acid to anti-PD-1 and anti-CTLA-4 facilitated NK cell infiltration and survival in mice bearing intracranial GL261 tumors (95, 96). Combining these approaches with techniques to prolong persistence and enhance cytolytic potential of adoptively transferred NK cells may assist in overcoming the BBB and the immunosuppressive brain TME (97).

### Group 1 Innate Lymphoid Cells (ILC1)

ILC1 express the transcription factor T-bet and secrete IFN-γ in response to IL-12, IL-15, and IL-18, facilitating control of intracellular pathogens by classical macrophage activation (98) or limiting local viral replication (99) (Figure 1A). It has been challenging to distinguish tissue ILC1s from NK cells because they share common functions and markers. The CNS contains NK cells, ILC1s, intermediate ILC1s, and “ex-ILC3” in the brain parenchyma, meninges, and choroid plexus (CP) (100). In contrast to CNS-NK cells, CNS-ILC1s are enriched in the CP (Figure 1B) (100). ILC1 functions in brain tumors awaits in depth evaluation but were investigated in the context of autoimmune encephalomyelitis (EAE) (100). Steady-state CNS-NK cells and CNS-ILC1s expressed similar amounts of IFN-γ, whilst CNS-ILC1s produced more TNF. In EAE, NK cells had increased IFN-γ and TNF expression whilst ILC1s maintained stable levels. Interestingly, CNS-ILC1s from naïve and EAE mice express granzyme B and perforin and degranulated suggesting anti-tumor cytotoxic potential.

NK cells, ILC1s, and intermediate ILC1s accumulated in the brain parenchyma as EAE progressed. Unlike NK cells, ILC1s and intermediate ILC1s did not proliferate *in situ*, suggesting entry into the brain parenchyma via meninges or CP. ILC1 distribution and response during EAE strongly suggests they can regulate neuroinflammation. CNS-ILC1 were CD49a^+^CD49b^−^ T-bet^+^Eomes^−^ whilst CNS-NK cells were CD49a^−^CD49b^+^ T-bet^+^Eomes^+^. These markers provide a useful foundation to study ILC1 and NK cell contributions to anti-tumor immunity in mouse models of brain cancer. The dynamics and cytotoxic potential of ILC1s observed in the context of EAE suggests a potential role for CNS-ILC1s in restricting the growth of a brain tumors by lysing tumor cells or secreting immunoregulatory cytokines (100). NKp44 is also expressed by human ILC1 and ILC3 and activated by PDGF-DD, a pathway implicated in greater GBM survival (Figure 1D). It will be interesting to delineate the relative contributions of human ILC1, ILC3, or NK cells to anti-tumor immunity in brain cancers, such as GBM (2).

### Group 2 Innate Lymphoid Cells (ILC2)

ILC2 express GATA3 and respond to IL-25, IL-33, and thymic stromal lymphopoietin (TSLP) by secreting IL-5, IL-9, and IL-13 to promote type II immunity (Figure 1A) (101). ILC2s are found in large numbers in the meninges of naïve mice enriched around the dural sinuses (Figure 1B). After spinal injury, meningeal ILC2s are activated by IL-33 to produce IL-5 and IL-13 and partially improve recovery following spinal cord injury (102). ILC2s accumulate in the CP of aged mice and represent the major lymphocyte subset in aged mice and humans (103). ILC2s in the aged brain produced large amounts of IL-5 and IL-13 in response to IL-33. Treatment with IL-5 or IL-33 or adoptive transfer of activated ILC2s into the brain improved neurogenesis and cognitive function by reducing TNF secretion from brain-resident CD8^+^ T cells. ILC2s may therefore play a neuroprotective role by orchestrating appropriate CNS immune responses (Figure 1D).

Many reports suggest type-II immunity downregulates anti-tumor immunity by hindering CTL. Mice genetically lacking ILC2s had markedly increased tumor growth rates and higher frequencies of circulating tumor cells and brain metastases (1,000-fold) (104). Tumor cell-derived IL-33 stimulated IL-13 secretion by ILC2s that enhanced DC antigen presentation and generation of anti-tumor CTL. The authors proposed a model where ILC2s mobilized from the lungs and other tissues enter tumors at distal sites to engage in immunosurveillance (104). It will be interesting to determine the relative contribution of meningeal ILC2s or ILC2s mobilized from tissues, such as the lungs, in restricting brain metastases and tumor growth (102). Interestingly, the primary male sex hormone testosterone, influenced ILC2 numbers and function and promoted and sustained a non-pathogenic TH2 myelin-specific response in EAE. These results suggest sexual dimorphism in ILC2 numbers or function could influence ILC2 brain tumor surveillance in addition to GBM invasiveness (105, 106). The protective role of activated ILC2s that observed during CNS injury, EAE, and restriction of brain metastases, suggests a potential role for activated meningeal ILC2s in suppressing brain tumor progression by enhancing CTL activity in response to elevated levels of IL-33 that are observed in brain cancers, such as glioma (107).

### Group 3 Innate Lymphoid Cells (ILC3)

ILC3 express RORγt and respond to IL-23 and IL-1β stimulation by secreting IL-17 and IL-22 that induce epithelial defense mechanisms and granulocytic responses to counteract extracellular bacterial and fungal infections (98) (Figure 1A). Lymphoid tissue-inducer (LTi) cells that trigger lymphoid tissue organogenesis during development are RORγt^+^ and produce IL-17 and IL-22 are categorized as ILC3 (Figure 1A) but emerge from the common lymphoid progenitor prior to ILCs (108). The role of ILC3 in brain cancer has not been extensively investigated. RORγt^+^ ILC3s are residents of the meninges in naïve B6 mice (109). LTi characteristically express c-Kit whereas expression of NKp46 differentiates ILC3 subtypes. A population of NKp46^+^CD4^+^ ILC3s was detected in the meninges but not in the CNS, whereas more c-Kit^+^ ILC3s were observed in the meninges than the CNS (Figure 1B).

In EAE, ILC3 numbers in the meninges and CNS increased. Meningeal ILC3s produced proinflammatory cytokines (IFN-γ, IL-17, and GM-CSF), and the costimulatory molecules (CD30L and OX40L) that promoted CD4^+^ memory T cell survival and function, which may impact the recognition of MHC class II-restricted neoantigens and response to immunotherapy TME (110). Moreover, c-Kit^+^ LTi cells, which can promote ectopic lymphoid follicle development, a hallmark of autoimmune diseases, were reduced in the meninges of EAE-resistant c-Kit mutant mice. Importantly, disease-induced trafficking of adoptively transferred wild type T lymphocytes to the meninges was impaired in ILC3-deficient *Rorc*^−/−^ mice showing ILC3s sustain neuroinflammation by supporting T cell survival and reactivation in the meninges (Figure 1D).

Another study found T-bet-dependent NKp46^+^ ILCs, which encompass NK cells, ILC1, and the NKp46 subset of ILC3s, were localized to the meninges and produced various inflammatory mediators that disrupted the BBB and facilitated infiltration of myelin-reactive TH17 cells into the brain parenchyma (111). Selective loss of T-bet in NKp46^+^ ILCs resulted in the reduction of NK cells and ILC1s in the meninges and production of IFN-γ by NKp46^+^ ILC3s, which impaired TH17 invasion of the CNS and protected from EAE disease. Importantly, NK cell-sufficient and NK cell-deficient mice showed similar levels of paralysis, suggesting NK cells do not play a major role in EAE immunopathogenesis and the pathogenic role of T-bet maps to the NKp46^+^ ILC3s and/or ILC1. Collectively, these findings suggest meningeal ILC3s could promote anti-tumor immunity in brain cancer by facilitating the infiltration of T lymphocytes into the brain and supporting their survival. It will be interesting to determine if the NKp44^+^ cells located in the brain parenchyma and associated with improved survival in GBM are derived from meningeal ILC3 populations or from other regions of the brain (2) (Figure 1D).

## Concluding Remarks

Transition of low-grade glioma to high-grade glioma, including transition to GBM, as well as post-treatment relapse remain major causes of treatment failure, and improved strategies to eradicate brain tumor cells are required. Understanding the functions of NK cells and ILCs in both healthy and tumorigenic brain is necessary for developing strategies for effective immunotherapy, including enhanced activation of brain resident NK cells and ILCs and transfusion and mobilization of engineered NK cells, e.g., CAR NK (112). Existing evidence, presented in this review, demonstrates a rapidly evolving NK cell and ILC research field and proposes that continued research efforts will lead to the development and refinement of NK- and ILC-based therapies which can be used in combination with existing standard and novel oncolytic virus and drug-based therapies to meaningfully enhance brain cancer patient outcomes.

## Author Contributions

AS, NG, and PC drafted the manuscript. TM and AB critically reviewed the manuscript for important intellectual content and approved it for publication. All authors contributed to the article and approved the submitted version.

## Conflict of Interest

The authors declare that the research was conducted in the absence of any commercial or financial relationships that could be construed as a potential conflict of interest.

## References

[1] Leece R Xu J Ostrom QT Chen Y Kruchko C Barnholtz-Sloan JS. Global incidence of malignant brain and other central nervous system tumors by histology, 2003-2007. Neuro Oncol. (2017) 19:1553–64. 10.1093/neuonc/nox091 28482030 PMC5737839

[2] Barrow AD Edeling MA Trifonov V Luo J Goyal P Bohl B. Natural killer cells control tumor growth by sensing a growth factor. Cell. (2018) 172:534–48.e19. 10.1016/j.cell.2017.11.037 29275861 PMC6684025

[3] Hsu J Hodgins JJ Marathe M Nicolai CJ Bourgeois-Daigneault M-C Trevino TN. Contribution of NK cells to immunotherapy mediated by PD-1/PD-L1 blockade. J Clin Invest. (2018) 128:4654–68. 10.1172/JCI99317 30198904 PMC6159991

[4] Korin B Ben-Shaanan TL Schiller M Dubovik T Azulay-Debby H Boshnak NT. High-dimensional, single-cell characterization of the brain's immune compartment. Nat Neurosci. (2017) 20:1300–9. 10.1038/nn.4610 28758994

[5] Lepennetier G Hracsko Z Unger M Van Griensven M Grummel V Krumbholz M. Cytokine and immune cell profiling in the cerebrospinal fluid of patients with neuro-inflammatory diseases. J Neuroinflammation. (2019) 16:219. 10.1186/s12974-019-1601-6 31727097 PMC6857241

[6] Kastrukoff LF Lau AS Takei F Carbone FR Scalzo AA. A NK complex-linked locus restricts the spread of herpes simplex virus type 1 in the brains of C57BL/6 mice. Immunol Cell Biol. (2015) 93:877–84. 10.1038/icb.2015.54 25971711

[7] Owens GC Garcia AJ Mochizuki AY Chang JW Reyes SD Salamon N. Evidence for innate and adaptive immune responses in a cohort of intractable pediatric epilepsy surgery patients. Front Immunol. (2019) 10:121. 10.3389/fimmu.2019.00121 30761153 PMC6362260

[8] Zhang Y Gao Z Wang D Zhang T Sun B Mu L. Accumulation of natural killer cells in ischemic brain tissues and the chemotactic effect of IP-10. J Neuroinflammation. (2014) 11:79. 10.1186/1742-2094-11-79 24742325 PMC4039314

[9] Liu Q Sanai N Jin W-N La Cava A Van Kaer L Shi F-D. Neural stem cells sustain natural killer cells that dictate recovery from brain inflammation. Nat Neurosci. (2016) 19:243–52. 10.1038/nn.4211 26752157 PMC5336309

[10] Ren F Zhao Q Huang L Zheng Y Li L He Q. The R132H mutation in IDH1 promotes the recruitment of NK cells through CX3CL1/CX3CR1 chemotaxis and is correlated with a better prognosis in gliomas. Immunol Cell Biol. (2019) 97:457–69. 10.1111/imcb.12225 30575118

[11] Holl EK Frazier VN Landa K Beasley GM Hwang ES Nair SK. Examining peripheral and tumor cellular immunome in patients with cancer. Front Immunol. (2019) 10:1767. 10.3389/fimmu.2019.01767 31417550 PMC6685102

[12] Yang I Han SJ Sughrue ME Tihan T Parsa AT. Immune cell infiltrate differences in pilocytic astrocytoma and glioblastoma: evidence of distinct immunological microenvironments that reflect tumor biology. J Neurosurg. (2011) 115:505–11. 10.3171/2011.4.JNS101172 21663411

[13] Domingues PH Teodósio C Ortiz J Sousa P Otero A Maillo A. Immunophenotypic identification and characterization of tumor cells and infiltrating cell populations in meningiomas. Am J Pathol. (2012) 181:1749–61. 10.1016/j.ajpath.2012.07.033 22982440

[14] Domingues P González-Tablas M Otero Á Pascual D Miranda D Ruiz L. Tumor infiltrating immune cells in gliomas and meningiomas. Brain Behav Immun. (2016) 53:1–5. 10.1016/j.bbi.2015.07.019 26216710

[15] Sivori S Parolini S Marcenaro E Castriconi R Pende D Millo R. Involvement of natural cytotoxicity receptors in human natural killer cell-mediated lysis of neuroblastoma and glioblastoma cell lines. J Neuroimmunol. (2000) 107:220–5. 10.1016/S0165-5728(00)00221-6 10854660

[16] Castriconi R Daga A Dondero A Zona G Poliani PL Melotti A. NK cells recognize and kill human glioblastoma cells with stem cell-like properties. J Immunol Baltim Md 1950. (2009) 182:3530–9. 10.4049/jimmunol.0802845 19265131

[17] Baker GJ Chockley P Yadav VN Doherty R Ritt M Sivaramakrishnan S. Natural killer cells eradicate galectin-1-deficient glioma in the absence of adaptive immunity. Cancer Res. (2014) 74:5079–90. 10.1158/0008-5472.CAN-14-1203 25038230 PMC4184887

[18] Haspels HN Rahman MA Joseph JV Gras Navarro A Chekenya M. Glioblastoma stem-like cells are more susceptible than differentiated cells to natural killer cell lysis mediated through killer immunoglobulin-like receptors-human leukocyte antigen ligand mismatch and activation receptor-ligand interactions. Front Immunol. (2018) 9:1345. 10.3389/fimmu.2018.01345 29967607 PMC6015895

[19] Castriconi R Dondero A Negri F Bellora F Nozza P Carnemolla B. Both CD133^+^ and CD133^−^ medulloblastoma cell lines express ligands for triggering NK receptors and are susceptible to NK-mediated cytotoxicity. Eur J Immunol. (2007) 37:3190–6. 10.1002/eji.200737546 17918205

[20] George RE Loudon WG Moser RP Bruner JM Steck PA Grimm EA. *In vitro* cytolysis of primitive neuroectodermal tumors of the posterior fossa (medulloblastoma) by lymphokine-activated killer cells. J Neurosurg. (1988) 69:403–9. 10.3171/jns.1988.69.3.0403 3261328

[21] Kennis BA Michel KA Brugmann WB Laureano A Tao R-H Somanchi SS Monitoring of intracerebellarly-administered natural killer cells with fluorine-19 MRI. J Neurooncol. (2019) 142:395–407. 10.1007/s11060-019-03091-5 30788681 PMC11492566

[22] Gras Navarro A Kmiecik J Leiss L Zelkowski M Engelsen A Bruserud Ø. NK cells with KIR2DS2 immunogenotype have a functional activation advantage to efficiently kill glioblastoma and prolong animal survival. J Immunol Baltim Md 1950. (2014) 193:6192–206. 10.4049/jimmunol.1400859 25381437 PMC4259203

[23] Dominguez-Valentin M Gras Navarro A Rahman AM Kumar S Retière C Ulvestad E. Identification of a natural killer cell receptor allele that prolongs survival of cytomegalovirus-positive glioblastoma patients. Cancer Res. (2016) 76:5326–36. 10.1158/0008-5472.CAN-16-1162 27406829

[24] Krenzlin H Behera P Lorenz V Passaro C Zdioruk M Nowicki MO. Cytomegalovirus promotes murine glioblastoma growth via pericyte recruitment and angiogenesis. J Clin Invest. (2019) 130:1671–83. 10.1172/JCI123375 30855281 PMC6436878

[25] Vauléon E Tony A Hamlat A Etcheverry A Chiforeanu DC Menei P. Immune genes are associated with human glioblastoma pathology and patient survival. BMC Med Genomics. (2012) 5:41. 10.1186/1755-8794-5-41 22980038 PMC3507656

[26] Zhong Q-Y Fan E-X Feng G-Y Chen Q-Y Gou X-X Yue G-J. A gene expression-based study on immune cell subtypes and glioma prognosis. BMC Cancer. (2019) 19:1116. 10.1186/s12885-019-6324-7 31729963 PMC6858694

[27] Lu J Li H Chen Z Fan L Feng S Cai X. Identification of 3 subpopulations of tumor-infiltrating immune cells for malignant transformation of low-grade glioma. Cancer Cell Int. (2019) 19:265. 10.1186/s12935-019-0972-1 31632199 PMC6788075

[28] Bockmayr M Klauschen F Maire CL Rutkowski S Westphal M Lamszus K. Immunologic profiling of mutational and transcriptional subgroups in pediatric and adult high-grade gliomas. Cancer Immunol Res. (2019) 7:1401–11. 10.1158/2326-6066.CIR-18-0939 31266783

[29] Zhu C Zou C Guan G Guo Q Yan Z Liu T. Development and validation of an interferon signature predicting prognosis and treatment response for glioblastoma. Oncoimmunology. (2019) 8:e1621677. 10.1080/2162402X.2019.1621677 31428519 PMC6685507

[30] Lee SJ Kang WY Yoon Y Jin JY Song HJ Her JH. Natural killer (NK) cells inhibit systemic metastasis of glioblastoma cells and have therapeutic effects against glioblastomas in the brain. BMC Cancer. (2015) 15:1011. 10.1186/s12885-015-2034-y 26704632 PMC4690248

[31] Baker GJ Chockley P Zamler D Castro MG Lowenstein PR. Natural killer cells require monocytic Gr-1(+)/CD11b(+) myeloid cells to eradicate orthotopically engrafted glioma cells. Oncoimmunology. (2016) 5:e1163461. 10.1080/2162402X.2016.1163461 27471637 PMC4938363

[32] Mostafa H Pala A Högel J Hlavac M Dietrich E Westhoff MA. Immune phenotypes predict survival in patients with glioblastoma multiforme. J Hematol OncolJ Hematol Oncol. (2016) 9:77. 10.1186/s13045-016-0272-3 27585656 PMC5009501

[33] Jiang T Wu W Zhang H Zhang X Zhang D Wang Q. High expression of B7-H6 in human glioma tissues promotes tumor progression. Oncotarget. (2017) 8:37435–47. 10.18632/oncotarget.16391 28415577 PMC5514920

[34] Tseng H-C Inagaki A Bui VT Cacalano N Kasahara N Man Y-G. Differential targeting of stem cells and differentiated glioblastomas by NK cells. J Cancer. (2015) 6:866–76. 10.7150/jca.11527 26284138 PMC4532984

[35] Kozlowska AK Tseng H-C Kaur K Topchyan P Inagaki A Bui VT. Resistance to cytotoxicity and sustained release of interleukin-6 and interleukin-8 in the presence of decreased interferon-γ after differentiation of glioblastoma by human natural killer cells. Cancer Immunol Immunother. (2016) 65:1085–97. 10.1007/s00262-016-1866-x 27439500 PMC4996719

[36] Kozlowska AK Topchyan P Kaur K Tseng H-C Teruel A Hiraga T. Differentiation by NK cells is a prerequisite for effective targeting of cancer stem cells/poorly differentiated tumors by chemopreventive and chemotherapeutic drugs. J Cancer. (2017) 8:537–54. 10.7150/jca.15989 28367234 PMC5370498

[37] Ruscetti M Leibold J Bott MJ Fennell M Kulick A Salgado NR. NK cell-mediated cytotoxicity contributes to tumor control by a cytostatic drug combination. Science. (2018) 362:1416–22. 10.1126/science.aas9090 30573629 PMC6711172

[38] Razavi S-M Lee KE Jin BE Aujla PS Gholamin S Li G. Immune evasion strategies of glioblastoma. Front Surg. (2016) 3:11. 10.3389/fsurg.2016.00011 26973839 PMC4773586

[39] Luna JI Grossenbacher SK Sturgill IR Ames E Judge SJ Bouzid LA. Bortezomib augments natural killer cell targeting of stem-like tumor cells. Cancers. (2019) 11:85. 10.3390/cancers11010085 30646520 PMC6356940

[40] Yoo JY Jaime-Ramirez AC Bolyard C Dai H Nallanagulagari T Wojton J. Bortezomib treatment sensitizes oncolytic HSV-1-treated tumors to NK cell immunotherapy. Clin Cancer Res. (2016) 22:5265–76. 10.1158/1078-0432.CCR-16-1003 27390350 PMC5093037

[41] Weiss T Schneider H Silginer M Steinle A Pruschy M Polić B. NKG2D-dependent antitumor effects of chemotherapy and radiotherapy against glioblastoma. Clin Cancer Res Off J Am Assoc Cancer Res. (2018) 24:882–95. 10.1158/1078-0432.CCR-17-1766 29162646

[42] Suryadevara CM Riccione KA Samspon JH. Immunotherapy gone viral: bortezomib and oHSV enhance antitumor NK-cell activity. Clin Cancer Res. (2016) 22:5164–6. 10.1158/1078-0432.CCR-16-1666 27521450 PMC5093093

[43] Han J Chen X Chu J Xu B Meisen WH Chen L. TGFβ treatment enhances glioblastoma virotherapy by inhibiting the innate immune response. Cancer Res. (2015) 75:5273–82. 10.1158/0008-5472.CAN-15-0894 26631269 PMC4681611

[44] Rossi GR Trindade ES Souza-Fonseca-Guimaraes F. Tumor microenvironment-associated extracellular matrix components regulate NK cell function. Front Immunol. (2020) 11:73. 10.3389/fimmu.2020.00073 32063906 PMC7000552

[45] Friese MA Wischhusen J Wick W Weiler M Eisele G Steinle A. RNA interference targeting transforming growth factor-beta enhances NKG2D-mediated antiglioma immune response, inhibits glioma cell migration and invasiveness, and abrogates tumorigenicity *in vivo*. Cancer Res. (2004) 64:7596–603. 10.1158/0008-5472.CAN-04-1627 15492287

[46] Powell AB Yadavilli S Saunders D Van Pelt S Chorvinsky E Burga RA. Medulloblastoma rendered susceptible to NK-cell attack by TGFβ neutralization. J Transl Med. (2019) 17:321. 10.1186/s12967-019-2055-4 31547819 PMC6757414

[47] Swann JB Hayakawa Y Zerafa N Sheehan KCF Scott B Schreiber RD. Type I IFN contributes to NK cell homeostasis, activation, and antitumor function. J Immunol Baltim Md 1950. (2007) 178:7540–9. 10.4049/jimmunol.178.12.7540 17548588

[48] Liu C Lou Y Lizée G Qin H Liu S Rabinovich B. Plasmacytoid dendritic cells induce NK cell-dependent, tumor antigen-specific T cell cross-priming and tumor regression in mice. J Clin Invest. (2008) 118:1165–75. 10.1172/JCI33583 18259609 PMC2230660

[49] Jarry U Donnou S Vincent M Jeannin P Pineau L Fremaux I. Treg depletion followed by intracerebral CpG-ODN injection induce brain tumor rejection. J Neuroimmunol. (2014) 267:35–42. 10.1016/j.jneuroim.2013.12.005 24369298

[50] Ursu R Carpentier A Metellus P Lubrano V Laigle-Donadey F Capelle L. Intracerebral injection of CpG oligonucleotide for patients with de novo glioblastoma-A phase II multicentric, randomised study. Eur J Cancer. (2017) 73:30–7. 10.1016/j.ejca.2016.12.003 28142059

[51] Rosen DB Bettadapura J Alsharifi M Mathew PA Warren HS Lanier LL. Cutting edge: lectin-like transcript-1 is a ligand for the inhibitory human NKR-P1A receptor. J Immunol Baltim Md 1950. (2005) 175:7796–9. 10.4049/jimmunol.175.12.7796 16339513

[52] Carpentier A Metellus P Ursu R Zohar S Lafitte F Barrie M. Intracerebral administration of CpG oligonucleotide for patients with recurrent glioblastoma: a phase II study. Neuro Oncol. (2010) 12:401–8. 10.1093/neuonc/nop047 20308317 PMC2940609

[53] Alizadeh D Zhang L Brown CE Farrukh O Jensen MC Badie B. Induction of anti-glioma natural killer cell response following multiple low-dose intracerebral CpG therapy. Clin Cancer Res Off J Am Assoc Cancer Res. (2010) 16:3399–408. 10.1158/1078-0432.CCR-09-3087 20570924 PMC3022005

[54] Burger MC Zhang C Harter PN Romanski A Strassheimer F Senft C. CAR-engineered NK cells for the treatment of glioblastoma: turning innate effectors into precision tools for cancer immunotherapy. Front Immunol. (2019) 10:2683. 10.3389/fimmu.2019.02683 31798595 PMC6868035

[55] Han J Chu J Chan WK Zhang J Wang Y Cohen JB. CAR-engineered NK cells targeting wild-type EGFR and EGFRvIII enhance killing of glioblastoma and patient-derived glioblastoma stem cells. Sci Rep. (2015) 5:11483. 10.1038/srep11483 26155832 PMC4496728

[56] Müller N Michen S Tietze S Töpfer K Schulte A Lamszus K. Engineering NK cells modified with an EGFRvIII-specific chimeric antigen receptor to overexpress CXCR4 improves immunotherapy of CXCL12/SDF-1α-secreting glioblastoma. J Immunother Hagerstown Md 1997. (2015) 38:197. 10.1097/CJI.0000000000000082 25962108 PMC4428685

[57] Poli A Wang J Domingues O Planagumà J Yan T Rygh CB. Targeting glioblastoma with NK cells and mAb against NG2/CSPG4 prolongs animal survival. Oncotarget. (2013) 4:1527. 10.18632/oncotarget.1291 24127551 PMC3824525

[58] Kmiecik J Gras Navarro A Poli A Planagumà JP Zimmer J Chekenya M. Combining NK cells and mAb9. 2.27 to combat NG2-dependent and anti-inflammatory signals in glioblastoma. Oncoimmunology. (2014) 3:e27185. 10.4161/onci.27185 24575382 PMC3916357

[59] Hallett WH Ames E Motarjemi M Barao I Shanker A Tamang DL. Sensitization of tumor cells to NK cell-mediated killing by proteasome inhibition. J Immunol. (2008) 180:163–70. 10.4049/jimmunol.180.1.163 18097016

[60] Zhang X Rao A Sette P Deibert C Pomerantz A Kim WJ. IDH mutant gliomas escape natural killer cell immune surveillance by downregulation of NKG2D ligand expression. Neuro-Oncol. (2016) 18:1402–12. 10.1093/neuonc/now061 27116977 PMC5035522

[61] Weiss T Weller M Guckenberger M Sentman CL Roth P. NKG2D-based CAR T cells and radiotherapy exert synergistic efficacy in glioblastoma. Cancer Res. (2018) 78:1031–43. 10.1158/0008-5472.CAN-17-1788 29222400

[62] Lanier LL. Up on the tightrope: natural killer cell activation and inhibition. Nat Immunol. (2008) 9:495–502. 10.1038/ni1581 18425106 PMC2669298

[63] Barrow AD Colonna M. The natural cytotoxicity receptors in health and disease. Front Immunol. (2019) 10:909. 10.3389/fimmu.2019.00909 31134055 PMC6514059

[64] Friese MA Platten M Lutz SZ Naumann U Aulwurm S Bischof F. MICA/NKG2D-mediated immunogene therapy of experimental gliomas. Cancer Res. (2003) 63:8996–9006. Available online at: https://cancerres.aacrjournals.org/content/63/24/8996 14695218

[65] Wischhusen J Friese MA Mittelbronn M Meyermann R Weller M. HLA-E protects glioma cells from NKG2D-mediated immune responses in vitro: implications for immune escape in vivo. J Neuropathol Exp Neurol. (2005) 64:523–8. 10.1093/jnen/64.6.523 15977644

[66] Robison NJ Yeo KK Berliner AP Malvar J Sheard MA Margol AS Phase I trial of dasatinib, lenalidomide, and temozolomide in children with relapsed or refractory central nervous system tumors. J Neurooncol. (2018) 138:199–207. 10.1007/s11060-018-2791-y 29427149 PMC5930136

[67] Soriani A Zingoni A Cerboni C Iannitto ML Ricciardi MR Di Gialleonardo V. ATM-ATR-dependent up-regulation of DNAM-1 and NKG2D ligands on multiple myeloma cells by therapeutic agents results in enhanced NK-cell susceptibility and is associated with a senescent phenotype. Blood. (2009) 113:3503–11. 10.1182/blood-2008-08-173914 19098271

[68] Gasser S Orsulic S Brown EJ Raulet DH. The DNA damage pathway regulates innate immune system ligands of the NKG2D receptor. Nature. (2005) 436:1186–90. 10.1038/nature03884 15995699 PMC1352168

[69] Fernández L Portugal R Valentín J Martín R Maxwell H González-Vicent M. *In vitro* natural killer cell immunotherapy for medulloblastoma. Front Oncol. (2013) 3:94. 10.3389/fonc.2013.00094 23626949 PMC3630393

[70] Gras Navarro A Espedal H Joseph JV Trachsel-Moncho L Bahador M Tore Gjertsen B. Pretreatment of glioblastoma with bortezomib potentiates natural killer cell cytotoxicity through TRAIL/DR5 mediated apoptosis and prolongs animal survival. Cancers. (2019) 11:996. 10.3390/cancers11070996 31319548 PMC6678126

[71] Meisen WH Kaur B. How can we trick the immune system into overcoming the detrimental effects of oncolytic viral therapy to treat glioblastoma? Expert Rev Neurother. (2013) 13:341–3. 10.1586/ern.13.25 23545048 PMC5298807

[72] Alvarez-Breckenridge CA Yu J Price R Wojton J Pradarelli J Mao H. NK cells impede glioblastoma virotherapy through NKp30 and NKp46 natural cytotoxicity receptors. Nat Med. (2012) 18:1827–34. 10.1038/nm.3013 23178246 PMC3668784

[73] Roy L-O Poirier M-B Fortin D. Transforming growth factor-beta and its implication in the malignancy of gliomas. Target Oncol. (2015) 10:1–14. 10.1007/s11523-014-0308-y 24590691

[74] Katz LH Li Y Chen J-S Muñoz NM Majumdar A Chen J. Targeting TGF-β signaling in cancer. Expert Opin Ther Targets. (2013) 17:743–60. 10.1517/14728222.2013.782287 23651053 PMC3745214

[75] Crane CA Han SJ Barry JJ Ahn BJ Lanier LL Parsa AT. TGF-β downregulates the activating receptor NKG2D on NK cells and CD8+ T cells in glioma patients. Neuro-Oncol. (2010) 12:7–13. 10.1093/neuonc/nop009 20150362 PMC2940557

[76] Beier CP Kumar P Meyer K Leukel P Bruttel V Aschenbrenner I. The cancer stem cell subtype determines immune infiltration of glioblastoma. Stem Cells Dev. (2012) 21:2753–61. 10.1089/scd.2011.0660 22676416 PMC3464079

[77] Close HJ Stead LF Nsengimana J Reilly KA Droop A Wurdak H. Expression profiling of single cells and patient cohorts identifies multiple immunosuppressive pathways and an altered NK cell phenotype in glioblastoma. Clin Exp Immunol. (2020) 200:33–44. 10.1111/cei.13403 31784984 PMC7066386

[78] Eisele G Wischhusen J Mittelbronn M Meyermann R Waldhauer I Steinle A. TGF-beta and metalloproteinases differentially suppress NKG2D ligand surface expression on malignant glioma cells. Brain J Neurol. (2006) 129:2416–25. 10.1093/brain/awl205 16891318

[79] Gao Y Souza-Fonseca-Guimaraes F Bald T Ng SS Young A Ngiow SF. Tumor immunoevasion by the conversion of effector NK cells into type 1 innate lymphoid cells. Nat Immunol. (2017) 18:1004–15. 10.1038/ni.3800 28759001

[80] Bollard CM Rössig C Calonge MJ Huls MH Wagner H-J Massague J. Adapting a transforming growth factor beta-related tumor protection strategy to enhance antitumor immunity. Blood. (2002) 99:3179–87. 10.1182/blood.V99.9.3179 11964281

[81] Yvon ES Burga R Powell A Cruz CR Fernandes R Barese C. Cord blood natural killer cells expressing a dominant negative TGF-β receptor: implications for adoptive immunotherapy for glioblastoma. Cytotherapy. (2017) 19:408–18. 10.1016/j.jcyt.2016.12.005 28109751

[82] Cedeno-Laurent F Watanabe R Teague JE Kupper TS Clark RA Dimitroff CJ. Galectin-1 inhibits the viability, proliferation, and Th1 cytokine production of nonmalignant T cells in patients with leukemic cutaneous T-cell lymphoma. Blood. (2012) 119:3534–8. 10.1182/blood-2011-12-396457 22383798 PMC3325040

[83] Hanihara M Kawataki T Oh-Oka K Mitsuka K Nakao A Kinouchi H. Synergistic antitumor effect with indoleamine 2,3-dioxygenase inhibition and temozolomide in a murine glioma model. J Neurosurg. (2016) 124:1594–601. 10.3171/2015.5.JNS141901 26636389

[84] Braud VM Allan DS O'Callaghan CA Söderström K D'Andrea A Ogg GS. HLA-E binds to natural killer cell receptors CD94/NKG2A, B and C. Nature. (1998) 391:795–9. 10.1038/35869 9486650

[85] Lee N Llano M Carretero M Ishitani A Navarro F López-Botet M. HLA-E is a major ligand for the natural killer inhibitory receptor CD94/NKG2A. Proc Natl Acad Sci U S A. (1998) 95:5199–204. 10.1073/pnas.95.9.5199 9560253 PMC20238

[86] Aldemir H Prod'homme V Dumaurier M-J Retiere C Poupon G Cazareth J. Cutting edge: lectin-like transcript 1 is a ligand for the CD161 receptor. J Immunol Baltim Md 1950. (2005) 175:7791–5. 10.4049/jimmunol.175.12.7791 16339512

[87] Roth P Mittelbronn M Wick W Meyermann R Tatagiba M Weller M. Malignant glioma cells counteract antitumor immune responses through expression of lectin-like transcript-1. Cancer Res. (2007) 67:3540–4. 10.1158/0008-5472.CAN-06-4783 17440061

[88] André P Denis C Soulas C Bourbon-Caillet C Lopez J Arnoux T. Anti-NKG2A mAb is a checkpoint inhibitor that promotes anti-tumor immunity by unleashing both T and NK cells. Cell. (2018) 175:1731–43.e13. 10.1016/j.cell.2018.10.014 30503213 PMC6292840

[89] Meng X Zhao Y Han B Zha C Zhang Y Li Z. Dual functionalized brain-targeting nanoinhibitors restrain temozolomide-resistant glioma via attenuating EGFR and MET signaling pathways. Nat Commun. (2020) 11:594. 10.1038/s41467-019-14036-x 32001707 PMC6992617

[90] Honda K Ohba Y Yanai H Negishi H Mizutani T Takaoka A. Spatiotemporal regulation of MyD88-IRF-7 signalling for robust type-I interferon induction. Nature. (2005) 434:1035–40. 10.1038/nature03547 15815647

[91] Carpentier A Laigle-Donadey F Zohar S Capelle L Behin A Tibi A. Phase 1 trial of a CpG oligodeoxynucleotide for patients with recurrent glioblastoma. Neuro Oncol. (2006) 8:60–6. 10.1215/S1522851705000475 16443949 PMC1871923

[92] Zhang C Oberoi P Oelsner S Waldmann A Lindner A Tonn T. Chimeric antigen receptor-engineered NK-92 cells: an off-the-shelf cellular therapeutic for targeted elimination of cancer cells and induction of protective antitumor immunity. Front Immunol. (2017) 8:533. 10.3389/fimmu.2017.00533 28572802 PMC5435757

[93] Reardon DA Gokhale PC Klein SR Ligon KL Rodig SJ Ramkissoon SH. Glioblastoma eradication following immune checkpoint blockade in an orthotopic, immunocompetent model. Cancer Immunol Res. (2016) 4:124–35. 10.1158/2326-6066.CIR-15-0151 26546453

[94] Taggart D Andreou T Scott KJ Williams J Rippaus N Brownlie RJ. Anti-PD-1/anti-CTLA-4 efficacy in melanoma brain metastases depends on extracranial disease and augmentation of CD8^+^ T cell trafficking. Proc Natl Acad Sci U S A. (2018) 115:E1540–9. 10.1073/pnas.1714089115 29386395 PMC5816160

[95] Galstyan A Markman JL Shatalova ES Chiechi A Korman AJ Patil R. Blood-brain barrier permeable nano immunoconjugates induce local immune responses for glioma therapy. Nat Commun. (2019) 10:1–13. 10.1038/s41467-019-11719-3 31462642 PMC6713723

[96] Ding H Portilla-Arias J Patil R Black KL Ljubimova JY Holler E. Distinct mechanisms of membrane permeation induced by two polymalic acid copolymers. Biomaterials. (2013) 34:217–25. 10.1016/j.biomaterials.2012.08.016 23063368 PMC3487713

[97] Barrow AD Colonna M. Tailoring natural killer cell immunotherapy to the tumour microenvironment. Semin Immunol. (2017) 31:30–6. 10.1016/j.smim.2017.09.001 28935344 PMC5659759

[98] Barrow AD Colonna M. Innate lymphoid cell sensing of tissue vitality. Curr Opin Immunol. (2018) 56:82–93. 10.1016/j.coi.2018.11.004 30529190 PMC6469350

[99] Weizman O-E Adams NM Schuster IS Krishna C Pritykin Y Lau C. ILC1 Confer early host protection at initial sites of viral infection. Cell. (2017) 171:795–808.e12. 10.1016/j.cell.2017.09.052 29056343 PMC5687850

[100] Romero-Suárez S Del Rio Serrato A Bueno RJ Brunotte-Strecker D Stehle C Figueiredo CA. The central nervous system contains ILC1s that differ from NK cells in the response to inflammation. Front Immunol. (2019) 10:2337. 10.3389/fimmu.2019.02337 31649664 PMC6795712

[101] Klose CSN Artis D. Innate lymphoid cells as regulators of immunity, inflammation and tissue homeostasis. Nat Immunol. (2016) 17:765–74. 10.1038/ni.3489 27328006

[102] Gadani SP Smirnov I Smith AT Overall CC Kipnis J. Characterization of meningeal type 2 innate lymphocytes and their response to CNS injury. J Exp Med. (2017) 214:285–96. 10.1084/jem.20161982 27994070 PMC5294864

[103] Fung ITH Sankar P Zhang Y Robison LS Zhao X D'Souza SS. Activation of group 2 innate lymphoid cells alleviates aging-associated cognitive decline. J Exp Med. (2020) 217:e20190915. 10.1084/jem.20190915 32022838 PMC7144523

[104] Saranchova I Han J Zaman R Arora H Huang H Fenninger F. Type 2 innate lymphocytes actuate immunity against tumours and limit cancer metastasis. Sci Rep. (2018) 8:2924. 10.1038/s41598-018-20608-6 29440650 PMC5811448

[105] Russi AE Ebel ME Yang Y Brown MA. Male-specific IL-33 expression regulates sex-dimorphic EAE susceptibility. Proc Natl Acad Sci U S A. (2018) 115:E1520–9. 10.1073/pnas.1710401115 29378942 PMC5816140

[106] Rodríguez-Lozano DC Piña-Medina AG Hansberg-Pastor V Bello-Alvarez C Camacho-Arroyo I. Testosterone promotes glioblastoma cell proliferation, migration, and invasion through androgen receptor activation. Front Endocrinol. (2019) 10:16. 10.3389/fendo.2019.00016 30778332 PMC6369181

[107] Zhang J-F Wang P Yan Y-J Li Y Guan M-W Yu J-J. IL-33 enhances glioma cell migration and invasion by upregulation of MMP2 and MMP9 via the ST2-NF-κB pathway. Oncol Rep. (2017) 38:2033–42. 10.3892/or.2017.5926 28849217 PMC5652951

[108] Ishizuka IE Chea S Gudjonson H Constantinides MG Dinner AR Bendelac A. Single-cell analysis defines the divergence between the innate lymphoid cell lineage and lymphoid tissue-inducer cell lineage. Nat Immunol. (2016) 17:269–76. 10.1038/ni.3344 26779601 PMC4755916

[109] Hatfield JK Brown MA. Group 3 innate lymphoid cells accumulate and exhibit disease-induced activation in the meninges in EAE. Cell Immunol. (2015) 297:69–79. 10.1016/j.cellimm.2015.06.006 26163773

[110] Alspach E Lussier DM Miceli AP Kizhvatov I DuPage M Luoma AM. MHC-II neoantigens shape tumour immunity and response to immunotherapy. Nature. (2019) 574:696–701. 10.1038/s41586-019-1671-8 31645760 PMC6858572

[111] Kwong B Rua R Gao Y Flickinger J Wang Y Kruhlak MJ T-bet-dependent NKp46^+^ innate lymphoid cells regulate the onset of TH17-induced neuroinflammation. Nat Immunol. (2017) 18:1117–27. 10.1038/ni.3816 28805812 PMC5605431

[112] Barrow AD Colonna M. Exploiting NK cell surveillance pathways for cancer therapy. Cancers. (2019) 11:55. 10.3390/cancers11010055 30626155 PMC6356551

